# Exogenous Putrescine Enhances Salt Tolerance and Ginsenosides Content in Korean Ginseng (*Panax ginseng* Meyer) Sprouts

**DOI:** 10.3390/plants10071313

**Published:** 2021-06-28

**Authors:** Md. Jahirul Islam, Byeong Ryeol Ryu, Md. Obyedul Kalam Azad, Md. Hafizur Rahman, Md. Soyel Rana, Jung-Dae Lim, Young-Seok Lim

**Affiliations:** 1 Department of Bio-Health Convergence, College of Biomedical Science, Kangwon National University, Chuncheon 24341, Korea; jahirulislam213@gmail.com (M.J.I.); fbqudfuf0419@naver.com (B.R.R.); azadokalam@gmail.com (M.O.K.A.); hafizknu94@gmail.com (M.H.R.); soyelrana98@gmail.com (M.S.R.); 2 Physiology and Sugar Chemistry Division, Bangladesh Sugarcrop Research Institute, Ishurdi 6620, Pabna, Bangladesh

**Keywords:** putrescine, *Panax ginseng*, ginseng sprouts, reactive oxygen species, salinity tolerance, protopanaxadiol, protopanaxatriol, ginsenosides

## Abstract

The effect of exogenously applied putrescine (Put) on salt stress tolerance was investigated in *Panax ginseng*. Thirty-day-old ginseng sprouts were grown in salinized nutrient solution (150 mM NaCl) for five days, while the control sprouts were grown in nutrients solution. Putrescine (0.3, 0.6, and 0.9 mM) was sprayed on the plants once at the onset of salinity treatment, whereas control plants were sprayed with water only. Ginseng seedlings tested under salinity exhibited reduced plant growth and biomass production, which was directly interlinked with reduced chlorophyll and chlorophyll fluorescence due to higher reactive oxygen species (hydrogen peroxide; H_2_O_2_) and lipid peroxidation (malondialdehyde; MDA) production. Application of Put enhanced accumulation of proline, total soluble carbohydrate, total soluble sugar and total soluble protein. At the same time, activities of antioxidant enzymes like superoxide dismutase, catalase, ascorbate peroxidase, guaiacol peroxidase in leaves, stems, and roots of ginseng seedlings were increased. Such modulation of physio-biochemical processes reduced the level of H_2_O_2_ and MDA, which indicates a successful adaptation of ginseng seedlings to salinity stress. Moreover, protopanaxadiol (PPD) ginsenosides enhanced by both salinity stress and exogenous Put treatment. On the other hand, protopanaxatriol (PPT) ginsenosides enhanced in roots and reduced in leaves and stems under salinity stress condition. In contrast, they enhanced by exogenous Put application in all parts of the plants for most cases, also evidenced by principal component analysis. Collectively, our findings provide an important prospect for the use of Put in modulating salinity tolerance and ginsenosides content in ginseng sprouts.

## 1. Introduction

Salinity is a major abiotic stress factor that can influence crop growth, yield, and quality [1]. The plants exposed to salinity experienced reducing water availability and ion accumulation, bringing in an imbalance of minerals. This inconsistency in ion accumulation is responsible for morphological, physiological, and metabolic disfunction in plants resulting in growth retardation [2]. Generally, a high concentration of salt ions increases the osmotic potential in soil, resulting in a physiological water shortage and hampering nutrient absorption in plants. Plants also incur a loss to photosynthesis rate by high sodium ions in the shoot [3]. Although variable strategies are followed to cope with salt stress, most crops could not grow well under salinity stress conditions. Response of shoot tissues under salt stress got prime attention in most studies as minimizing toxic ions accumulation in leaf is essential for plant growth and development. However, roots are exposed to salt stress as the first organ and frequently have more significant growth retardation than shoots [4].

Plants adopt complex strategies by bringing in several physiological and biochemical changes for dealing with salt stress include osmotic adjustment, increasing antioxidant enzyme activities, toxic ion elimination, and compartmentalization, secondary metabolites synthesis and accumulation [3,5,6]. These adaptation strategies occurred simultaneously or separately, depending on the stress duration and intensity to combat salt stress in the long-term evolutionary process [3]. Although the accumulation of toxic ions in the leaf is crucial in the salt tolerance mechanism of the plant, retardation of root growth is the most critical change as it is the first organ exposed to salt stress [4]. In addition, plants under salt stress generate a pool of reactive oxygen species (ROS) like superoxide anion radical (O_2_^−•^), hydrogen peroxide (H_2_O_2_), and hydroxyl radical (OH^•^) [7]. These ROS affect the normal metabolism process through lipid peroxidation or oxidative damage of biological molecules resulting in an imbalance of component quantities and dysfunction of their typical defensive system [8,9]. Other studies have also demonstrated that young senescing leaf cells are more vulnerable and produce excessive ROS under abiotic stress, are subject to abolishment by a series of enzymatic and non-enzymatic activities [10]. In general, plants use antioxidant enzymes—including superoxide dismutase (SOD), catalase (CAT), ascorbate peroxidase (APX), and peroxidase (GPX) [11,12]—combined with low molecular weight antioxidants such as ascorbic acid (AsA) and glutathione (GSH) [13,14], and compatible solutes like proline (Pro), total soluble sugar (TSS), and total soluble carbohydrates (TSC) [8] to quench the intense flux of ROS. Therefore, ROS homeostasis by the combined action of enzymatic and non-enzymatic antioxidants plays a vital role in improving the salt tolerance of plants.

Korean ginseng (*Panax ginseng* Meyer) is one of the most important medicinal crops in East Asia, has been utilized as a tonic, stimulant, and to alleviate stress symptoms for over 2000 years [15,16]. The most common bioactive ingredients in ginseng include ginsenosides, polysaccharides, peptides, polyacetylenic alcohol, and fatty acids [17]. Among them, ginsenosides, also known as triterpene glycoside saponin, are most common and well known in pharmacological and physiological fields as a remedial of anticancer, antidiabetic, immunomodulatory, neuroprotective, radioprotective, antiamnestic, and antistress [16]. Earlier it was well described that ginsenosides content in ginseng plants varies depending on factors like light, water, temperature, ionizing radiation, soil, location, plant age, and so on [18,19,20]. Salt stress is also a crucial factor that affects the growth, development and yield of ginseng plants [21]. The amount of ginsenosides was also depicted higher in leaves than roots of ginseng plants in recent studies [22,23]. Importantly, ginseng sprouts have become a new medicinal vegetable according to the revised Ginseng Industry Act 2015 [24] which play a role in growing more consumer interest in ginseng sprout. 

Plants produce secondary metabolites that take part in several physiological roles; under stress condition regulation, production and, their roles in defense and tolerance mechanisms are well described [8,25]. The prime secondary metabolites of ginseng are under the group triterpene saponins, collectively known as ginsenosides [26]. Ginsenosides can be divided into dammarane and oleanane type saponins according to their skeletons [27,28]. The dammarane-type ginsenosides divided into two groups: protopanaxadiol (PPD) and protopanaxatriol (PPT) [29] that are produced to meet the demand of growth and defense purposes [16,30]. Therefore, modulation of ginsenosides under abiotic stress may have a crucial connection to the stress response of ginseng plants. In this connection, upregulation of ginsenosides by applying elicitor is also an attractive strategy nowadays [16]. Although many reports have been documented regarding the pharmacological effects of ginsenosides, little is known about the biosynthesis pathway, especially that regulated by stress response hormone. 

Polyamines (PAs) are the universal organic polycations that contain two or more amino groups and are affiliated with some fundamental physiological processes of plants like growth, development, and senescence [31]. PAs also can respond to biotic and abiotic stresses by adjusting osmosis and scavenging of ROS from the cell [32,33], thus regulating the normal physiological processes of plants [34,35]. Salinity affects morpho-physiological and metabolic processes by accumulating varied ROS and inhibiting the antioxidant enzyme activities in plants [2]. Exogenous PAs, including Put, protect plasma membrane by controlling MDA and other ROS, reduce Na^+^ accumulation, increase antioxidant activities and photosynthetic capacity of salinity affected plants [36]. In the PA biosynthesis pathway, putrescine (Put) is the central product and acts as a synthetic precursor of spermidine (Spd) and spermine (Spm). Among the three different biosynthetic routes, the primary Put synthesis follows the removal of nitrogen atoms from agmatine (Agm) to form N-carbamoyl Put (NCPA). After that, NCPA is hydrolyzed by N-carbamoylputrescine amidohydrolase (NCPAH) to form putrescine [35].

Controlled foliar application of Put can trigger physiological processes and induce osmotic adjustment molecules like proline, total soluble sugars and amino acids in plants [35]. The role of PAs in the biosynthesis of DNA, RNA, and protein, maintaining plant growth and development, lingering ageing, and protecting the membrane from oxidative damage by removing free radicals in plants were also reported [37]. Earlier, exogenous Put was reported enhanced salt tolerance by accumulating higher osmolytes, regulating carbon fixation, electron transport, energy and defence pathways in *Cynodon dactylon* [38]; eliminating reactive oxygen species in *Citrus aurantium* [39]; regulation of photosynthetic pathways in *Citrus reticulata* × *Citrus limetta* [40]; photosynthetic [41] and root growth [4] associated proteins in *Cucumis sativa*. In case of ginseng, the beneficial role of exogeneous Spd through preventing chlorophyll degradation, enhancing proline and activity of antioxidant enzymes was described under salinity stress condition [2]. However, many aspects of Put-mediated salinity stress tolerance in ginseng plants remains elusive. Here we provide the first report of the potential beneficial roles of exogenous Put in modulating salinity stress tolerance in ginseng sprouts by regulating physio-biochemical traits and ginsenosides accumulation.

## 2. Results and Discussion

### 2.1. Growth Parameters

The growth rate (GR) of salt-treated ginseng sprouts decreased severely, but the application of Put improved it compared to salinity stress condition. Ginseng sprouts treated by 0.6 mM Put was most effective in improving GR in salinity treatment. Similar results (Table 1) were also observed in shoot length (SL), shoot fresh weight (SFW), root fresh weight (RFW), and root dry weight (RDW). On the other hand, neither salinity stress nor Put treatments made any significant differences in root length (RL), root–shoot ratio (RSR), and shoot dry weight (SDW).

Put has various functions in all living organisms, and its positive influence under several abiotic stress has been well documented, but their exact nature of work on the plant under stress is still under debate [4,40,42,43]. It has been suggested that Put may be involved in several hormonal pathways, scavenging ROS, and adjust osmotic balance [44,45,46]. It was reported that salt stress causes more damage to plant aerial part than plant roots [47]. Wilting and necrosis in plant leaves are typical appearance under salt stress [3]. Our results showed that the growth status of ginseng aerial parts, RFW and RDW, are significantly affected by salt stress. Exogeneous spraying of Put maintained an excellent recovery of them under 0.6 mM concentration in GR, SFW, RFW, and RDW compared to salinity stress. This phenomenon reflects strong evidence of salt tolerance of ginseng plant with Put application. Although, it was demonstrated that root yield, including root length, root surface area, and root weight reduced under salinity stress [48,49]. However, RL and RSR did not show any significant response under salinity stress or in Put application, which is unclear in the present study. 

### 2.2. Changes in Chlorophyll Content and Fluorescence Parameters

**Figure 1:**
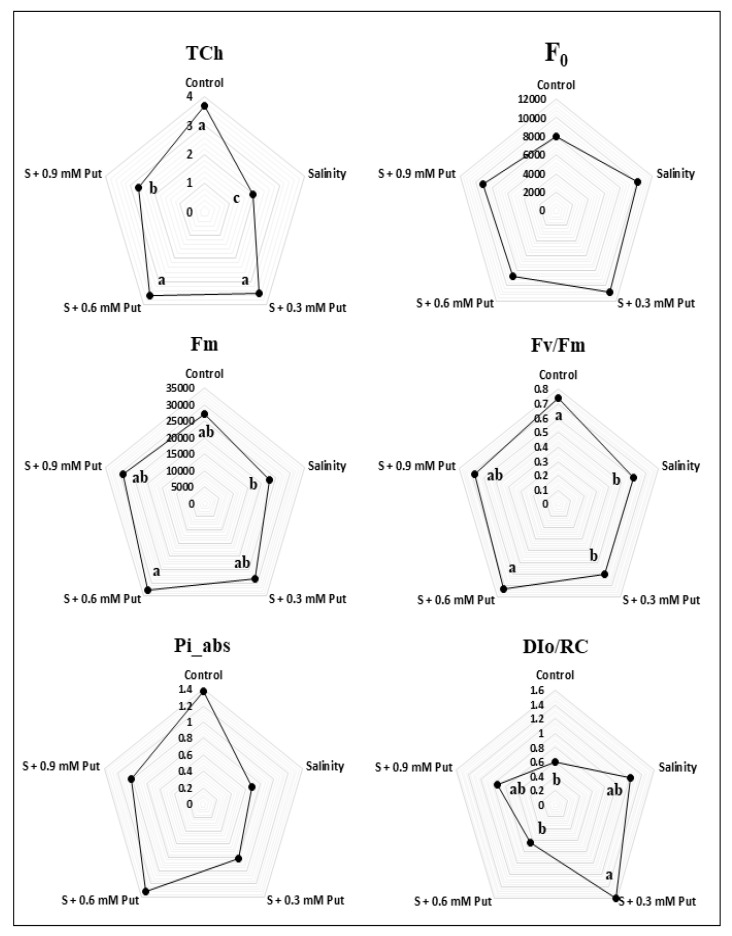
Effect of foliar application of putrescine on fluorescence intensity at 50 µs (F_0_), maximal fluorescence intensity (F_m_), maximum photosynthetic quantum yield (Fv/Fm), calculated PSII performance index (Pi_abs), and dissipated energy flux (DI_0_/RC), of ginseng seedlings grown under salinity stress condition.

Data presented in Figure 1 showed that the total chlorophyll (Tch) content was decreased under salinity stress compared to the control condition. Similarly, among the OJIP parameters, maximal fluorescence intensity (F_m_), maximum photosynthetic quantum yield (Fv/Fm), and calculated PSII performance index (Pi_abs) were found to be low in ginseng leaves under salinity conditions. On the other hand, fluorescence intensity at 50 µs (F_0_) and dissipated energy flux (DI_0_/RC) was increased under salinity stress condition. Application of Put at 0.6 mM concentration increased the Tch content, Fm, Fv/Fm, and Pi_abs considerably, while F_0_ and DI_0_/RC were decreased compared to salinity stress condition.

Photosynthetic pigments content decreased mainly due to rapid disruption and inhibition of synthesis [50]. In our study, the Tch content showed a clear declining trend in ginseng leaves (Figure 1). Oxidative stress enhances ROS accumulation which tends to accelerate chlorophyll degradation by producing lipid peroxidation and the instability of the pigment-protein complex in leaf tissue [2,42,51]. Exogenous Put application enhances photosynthetic pigments, and these processes are well documented in some previous studies [52,53]. In our study, spraying with Put at different concentrations improved Tch concentrations in ginseng leaves, where 0.6 mM concentration showed the best result. Earlier, the exogenous spermidine application was found to be enhanced photosynthetic pigments content in ginseng seedling grown under saline stress condition [2]. Putrescine plays a vital role protects thylakoid membranes through a chlorophyll–protein complex site and positively impacts chlorophyll levels [54].

Put treated plants exhibited a better performance of OJIP parameters as well as enhanced Tch under saline stress condition. These two positive effects may also have accounted for the enhanced plant growth of Put treated plants, most probably by enhancing CO_2_ fixation under salt stress. In this connection, several studies found a correlation between abiotic stress tolerance and efficiency of photosystem II [55,56]. In our study, salt stress significantly reduced the photosynthetic quantum yield (Fv/Fm) in ginseng plants, while it improved most with a 0.6 mM of Put application. Similarly, higher chlorophyll fluorescence parameters like F_0_, Fm, and Fv/Fm were reported by exogenous Put application in the Iranian mandarin Bakraii (*Citrus reticulata* × *Citrus limetta*) under salinity stress [40]. In some previous studies, higher Fv/Fm was also denoted as a stress tolerance indicator like cold stress [57,58], salinity stress [59,60], and drought stress [61,62]. Plants with higher Pi_abs [63] and lower DI_0_/RC [64] have also demonstrated high tolerance to oxidative stress. It is believed that under abiotic stress conditions, photosynthetic pigments of photosystems are damaged by stress factors resulting in a low light-absorbing efficiency in PS I and PS II. This reduced light-absorbing efficiency is the prime cause of reduced photosynthetic capacity in plants [65]. Our present study also supports this finding with lower Tch and photosynthetic capacity (photosynthetic quantum yield) in salt stress which was improved by exogenous Put application.

### 2.3. Changes in Osmotic Adjustment Molecules

**Figure 2:**
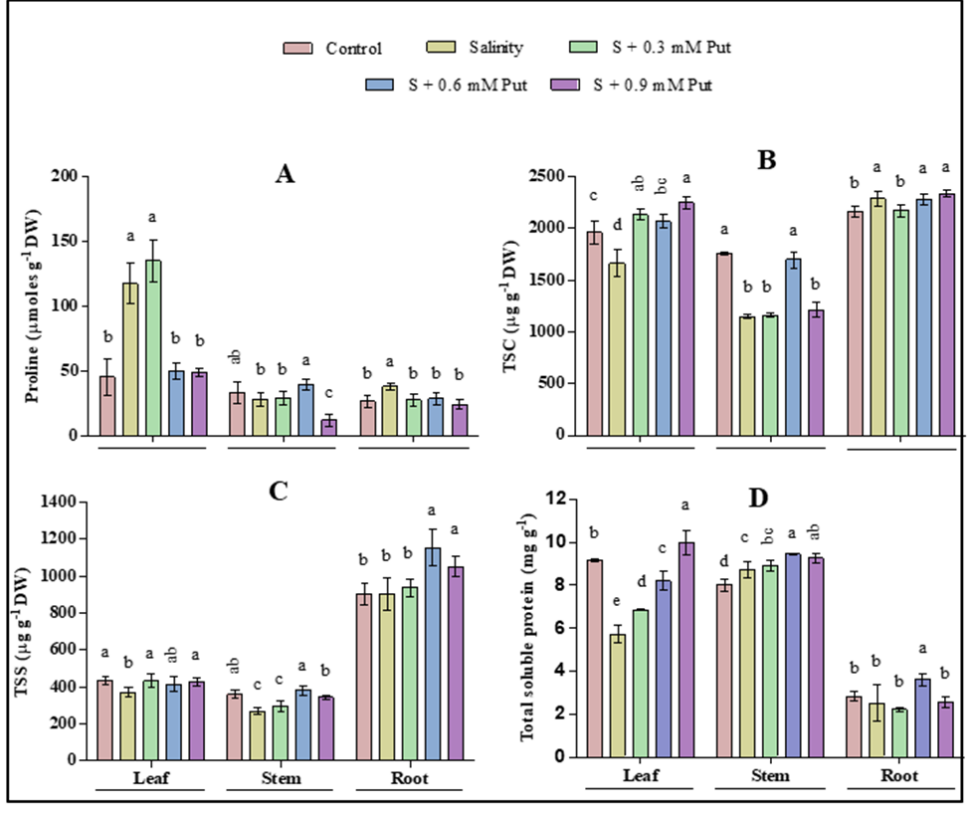
Effect of foliar application of putrescine on proline (**A**) Pro; total soluble carbohydrates (**B**), TSC; total soluble sugar (**C**), TSS; and total soluble protein (**D**), TSS of ginseng seedlings grown under salinity stress condition. Different letters indicate significant differences (*p* ≤ 0.05) among the treatments within each parameter. Each value represents the mean ± SD (*n* = 4).

Different responses were observed in leaf, stem, and root regarding proline (Pro), total soluble carbohydrate (TSC), total soluble sugar (TSS), and total soluble protein (TSP) content (Figure 2). Leaves and stems were affected more than other parts of the ginseng plant regarding TSC and TSS under salinity stress. Exogeneous Put treatment increased both TSC and TSS in all parts of plants. Proline significantly increased in leaves and roots where it reduced again after Put treatment (except 0.3 mM at leaves). In case of TSP, only leaves were found to significantly decreased under saline stress condition. Results also indicated that Put has a significant effect on accumulating TSP by foliar application of 0.9 mM and 0.6 mM concentration in leaves, and stems and roots of salinity-affected plants, respectively.

Compatible solutes which are known as osmotic adjustment molecules, maintain a strong correlation between their quantity and stress tolerance level in plants [66]. Generally, the ROS homeostasis process is affected by abiotic stress by stimulating overproduction resulting in oxidative damage of the plant. Pro is considered the main osmotic adjustment molecules with a low molecular weight that plays a crucial role in regulating redox potential [67], scavenging hydroxyl radical [68], thus mitigating oxidative damage and stabilizing the cell membrane [69] under stress condition. Pro accumulation is also an essential indicator for plant response to salt stress to protect from such damage [70]. In the present study, the leaves and roots of ginseng seedlings under salt stress showed a higher accumulation of proline than stem. In addition, a significant uprising was observed by the spraying of 0.3 mM, and 0.6 mM Put in leaves and stem, respectively. Therefore, Put treated seedlings accumulate more Pro, which indicates an adaptation to salinity [2,40,71]. 

Besides, amino acids play a pivotal role in protein biosynthesis. As a vital amino acid, Pro imparts stress tolerance by maintaining cell turgor or osmotic balance and preventing electrolyte leakage [66]. Our study also found a positive relation between Pro and TSP accumulation in leaves and stem tissue by exogenous Put treatment. The role of Pro, TSC, TSS, and TSP to avoid abiotic stress by improving osmotic adjustment, ROS detoxification, protein stabilization, and cell membrane protection was described in some previous study [8,72,73,74,75]. As an important osmotic adjustment component, TSC and TSS have also been narrated as closely associated with the antioxidation system at the cellular level, which actively participated in ROS detoxification [75]. A significant decrease of TSC was observed both in leaves and stems under salinity stress, where it was increased by the spraying of 0.3 mM and 0.6 mM Put, respectively. TSS decreased significantly only in leaf and stem under saline stress while it was increased by spraying 0.6 mM Put in all parts of the plant. A similar finding was also reported in wheat under drought stress, where variability was observed in the influence of exogenous Put on Pro, soluble, and insoluble sugar accumulation [76]. A previous study also reported decreased TSC under drought conditions in *Castanea sativa* [77]. Protein synthesis is considered as a possible primary target of salt toxicity due to the inhibition of potassium in the protein synthesis system by sodium and chloride [73], which may be controlled by some enzymatic system [78]. However, the response of soluble protein under salinity depends on species and variety. Soluble protein may act as osmotic adjustment molecules by accumulating or remain unchanged during salinity stress which can reutilize as nitrogen when stress is over [73,79]. It was also reported that soluble protein might be converted to free amino acids, thus helps to maintain the physiological function of plants under severe stress [75,80]. In our study, salt stress decreased TSP content in leaf tissue whereas increased in the stem of ginseng sprouts. However, no significant change was observed in root tissues. A decreased total soluble protein in the leaf of two strawberry cultivars named Tioga and Chandler was also reported earlier [73]. On the other hand, TSP accumulation was recovered by exogenous application of Put in leaves, stem, and root tissues. 

### 2.4. Malondialdehyde and H_2_O_2_ Concentration

**Figure 3:**
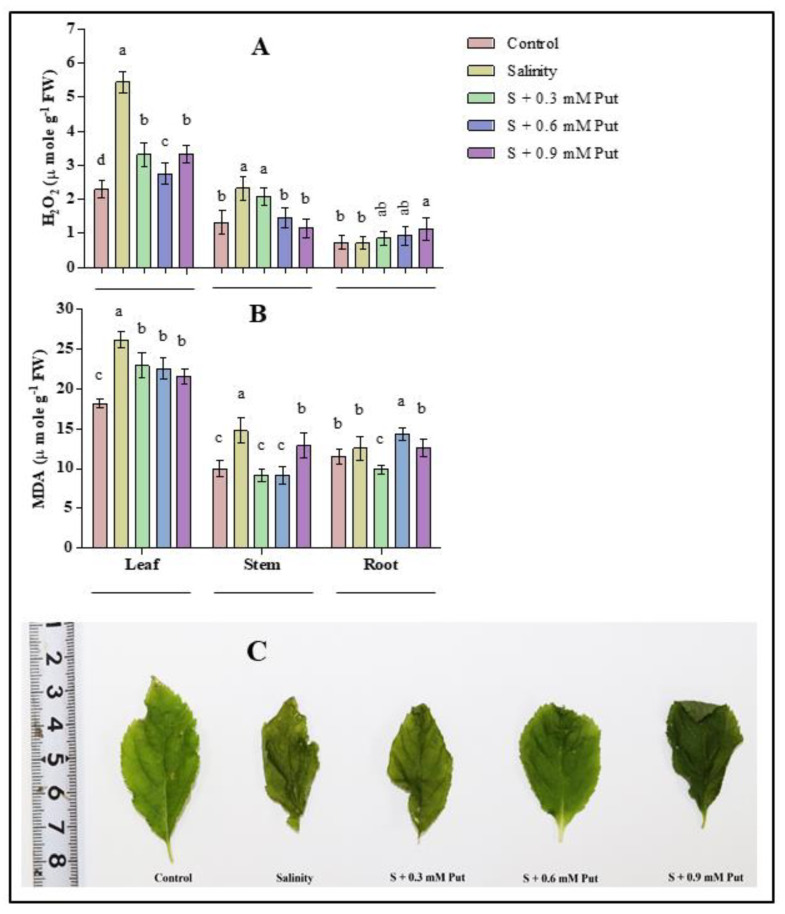
Effect of foliar application of putrescine on hydrogen peroxide (**A**) H_2_O_2_; malondialdehyde (**B**), MDA accumulation and leaf necrosis of ginseng seedlings grown under salinity stress condition (**C**). Different letters indicate significant differences (*p* ≤ 0.05) among the treatments within each parameter. Each value represents the mean ± SD (*n* = 4).

Salinity significantly changed the concentration of hydrogen peroxide (H_2_O_2_) (*p* ≤ 0.05) in the leaves (2.4 folds) and stems (1.76 folds) of ginseng seedlings compared to control (Figure 3A). The level of H_2_O_2_ decreased most with the treatment of 0.6 mM Put in leaves and stems under salinity stress condition. On the other hand, a very negligible effect was observed in roots for both under salinity and Put application.

The effect of salinity on lipid peroxidation or cellular damage indicator (malondialdehyde; MDA) is shown in Figure 3B. MDA levels were raised significantly (*p* ≤ 0.05) in leaf (1.44 folds) and stem (1.48 folds), whereas no change was observed in root under salinity stress conditions. On the other hand, the lipid peroxidation level decreased with a low level (0.3 mM) of exogenous Put application in leaf, stem, and root of ginseng sprouts. 

Salinity is well recognized for provoking ROS generation. Excess ROS is responsible for creating oxidative stress in plant cells where lipids and proteins are the primary victims [81]. Oxidative stress by any abiotic stress is mainly induced by interrupting electron flow in the photosynthesis process, primarily responsible for ROS generation [82]. It was narrated that oxidative stress varies among the plant tissues under salt stress, where root tissues are the prime victim compared to leaves [83]. In the present study, salinity stress substantially increased H_2_O_2_ and MDA in different parts of the ginseng plants. Among them, leaves and stems tissues showed more vulnerability to salinity than roots. This may be due to quick damage of photosynthetic pigments, fluorescence activities, and protein disruption in leaf tissues of ginseng seedlings compared to roots (Figure 3C). Generally, MDA levels, commonly used to determine the quantity of lipid peroxidation, was responsible for photosynthetic pigment disorganization, protein denaturation, enzyme activity inhibition, and finally, programmed cell death [81,84,85,86]. On the other hand, depending on the concentration and ROS scavenging mechanism, H_2_O_2_ can act as both a cell-damaging and signaling molecule [8,87,88]. Both MDA and H_2_O_2_ produced as a by-product of metabolic pathways are responsible for lowering plant growth and development [89], which entirely supports our findings.

**Figure 4:**
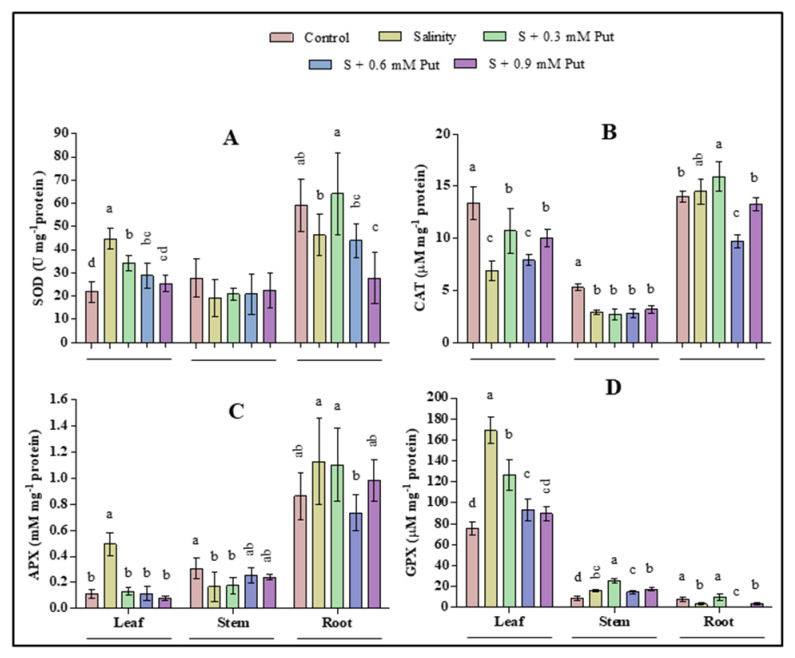
Effect of Putrescine on antioxidant enzymes (superoxide dismutase (**A**), SOD; catalase (**B**), CAT; ascorbate peroxidase (**C**), APX; and guaiacol peroxidase (**D**), GPX) activities in leaves of ginseng seedlings under salinity stress condition. Different letters indicate significant differences (*p* ≤ 0.05) among the treatments within each parameter. Each value represents the mean ± SD (*n* = 4).

In contrast, put application increased the osmolytes and the activities of antioxidant enzymes in some instances (Figure 4) and resulted in a lower MDA and H_2_O_2_ accumulation under salinity stress. Several earlier studies also reported the exogenous Put application that modulates plant growth and photosynthetic apparatus, stimulates antioxidants capacity and gene expression of plants under different abiotic stress [35,42,43,44,90]. Besides, exogenous Put at 0.9 mM concentration created chlorosis and necrosis in leaf tissue, which indicated a toxic effect of Put on ginseng sprouts. It was stated that overdose or a high concentration of Put might activate arginine decarboxylase (ADC) that further introduces K+ deficiency or osmotic shock, leading to leaf chlorosis, leaf necrosis, and shortened stem and roots [91].

### 2.5. Changes in Antioxidant Enzyme Activity

The effects of salinity and application of Put in different concentrations on antioxidant enzyme activity were shown in Figure 4. Compared with control, superoxide dismutase (SOD), ascorbate peroxidase (APX), and guaiacol peroxidase (GPX) activity increased significantly (*p* ≤ 0.05) under salinity stress in the case of leaves. The activity of catalase (CAT) significantly declined under salinity stress, where it further increased by 0.3 mM Put. Besides, the activities of SOD and GPX were also recorded higher by exogenous 0.3 mM Put application compared to control. In case of stem, the activities of CAT and APX enzymes significantly (*p* ≤ 0.05) decreased under salinity stress, whereas both salinity and 0.3 mM Put application significantly increased the activity of GPX. 

In case of root, the activity of SOD and GPX were significantly (*p* ≤ 0.05) decreased while no significant change was observed for CAT and APX under salinity stress. Besides, the application of Put at 0.3 mM concentration significantly improved the activities of both SOD and GPX compared to salinity stress condition.

Results of this study showed that SOD, APX, and GPX enzymes activity increased where CAT activity decreased in leaves of ginseng seedlings under salinity stress. Numerous studies have demonstrated that plants treated with NaCl increased the activities of SOD, APX, and GPX in leaves [92,93,94]. The activity of CAT decreased in leaves under salinity stress was also demonstrated by other studies [95,96,97]. Antioxidant enzyme defense activities have a high positive correlation with increased abiotic stress tolerance. SOD acts as a first-line ROS scavenger in such defense activities by catalyzing the superoxide radical (O_2_^−•^) into oxygen and H_2_O_2_ [8]. When plants are exposed to excess salinity, ROS accumulation is triggered, which impede the balance between ROS accumulation and the scavenging system [98]. This excess ROS may initiate membrane lipid peroxidation, deteriorate lipid unsaturation, enhance protein polymerization and membrane permeability resulting in cellular oxidative damage to plants [99,100]. Generally, excess superoxide generation enhances SOD activity, which can also act as a signal for accelerating antioxidant enzyme induction, resulting in more significant SOD generation [101,102]. Besides, CAT, APX, and GPX activity are primarily responsible for the detoxification of H_2_O_2_ to H_2_O and O_2_.

Moreover, excess ROS destruct the chloroplast envelope and thylakoid membrane and induce stomatal closure by decreasing the endogenous PAs in thylakoid membranes, which harm the plant by degrading photosynthetic pigments and PS II photochemistry [90]. The increased activity of SOD, APX, and GPX in leaves may result from a higher accumulation of ROS under salinity stress to combat and minimize cell damage. However, the application of Put at 0.3–0.6 mM concentration proved effective and maximally diminished the level of MDA and H_2_O_2_. This reduction in ROS generation seemed due to the increased Put-mediated activities of SOD, CAT, and GPX along with osmolytes. It was stated that exogenous PAs, including Put could enter into the chloroplast quickly and protect the photosynthetic apparatus from abiotic stresses [90,103]. The decreased ROS with higher enzymatic activities by exogenous Put application demonstrates a stable and comfortable situation in plants, clearly supported by the ameliorated morpho-physiological parameters. Overall, the enzymatic activities of the present study increased comparatively higher in leaves under salinity stress than that of stems and roots. These results indicated that leaves other than stems and roots are most responsive to salinity stress and play a major role in scavenging the ROS from ginseng plants to protect them from abiotic stress. 

### 2.6. Changes in Ginsenosides Accumulation

**Figure 5:**
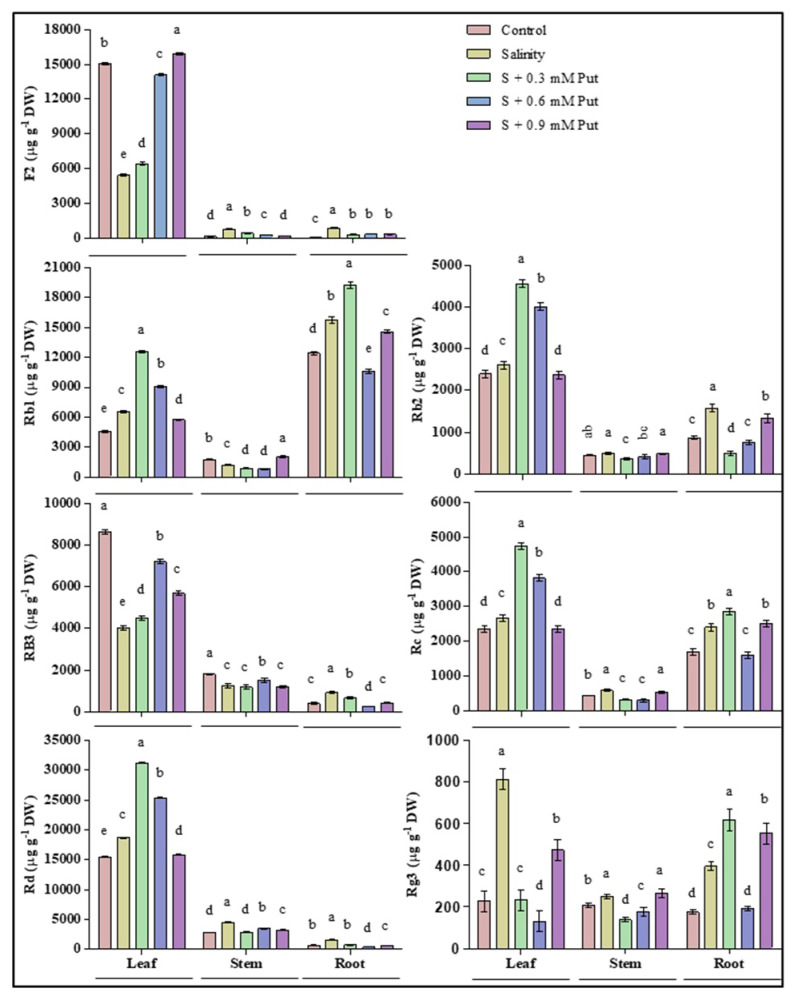
Effect of Putrescine on Ginsenosides (PPD type) in leaf, stem, and root of ginseng seedlings under salinity stress condition. Different letters indicate significant differences (*p* ≤ 0.05) among the treatments within each parameter. Each value represents the mean ± SD (*n* = 3).

The effects of salinity and application of Put in different concentrations of protopanaxadiol (PPD) types of ginsenosides in leaves, stems, and roots were shown in Figure 5. Results showed similarities not only in ginsenoside organ-specific distribution but also in fluctuation patterns at different treatments. F2, Rb2, Rb3, Re, and Rd were recorded as the primary ginsenosides in leaves, where Rb2, Re, and Rd increased, and F2 and Rb3 decreased significantly under salinity stress. Exogeneous Put application impressively upregulated the accumulation of leaves ginsenosides at 0.3 mM and 0.6 mM concentration level. Rb1 and Re were recorded as the primary ginsenosides of both leaves and roots, where significantly greater accumulation was observed under salinity stress. Besides, the highest accumulation of Rb1 and Re were recorded in spraying of 0.6 mM Put under salinity treated plants. On the other hand, Rg3 was found as the main ginsenoside in both leaves, stems, and roots of ginseng seedlings. Both salinity and Put at 0.9 mM concentration enhanced the Rg3 accumulation in plants. Unlike others, Put concentration at 0.3 mM to 0.6 mM level decreased the accumulation of Rg3 to a minimum level, and an opposite result was observed in that of 0.9 mM concentration.

**Figure 6:**
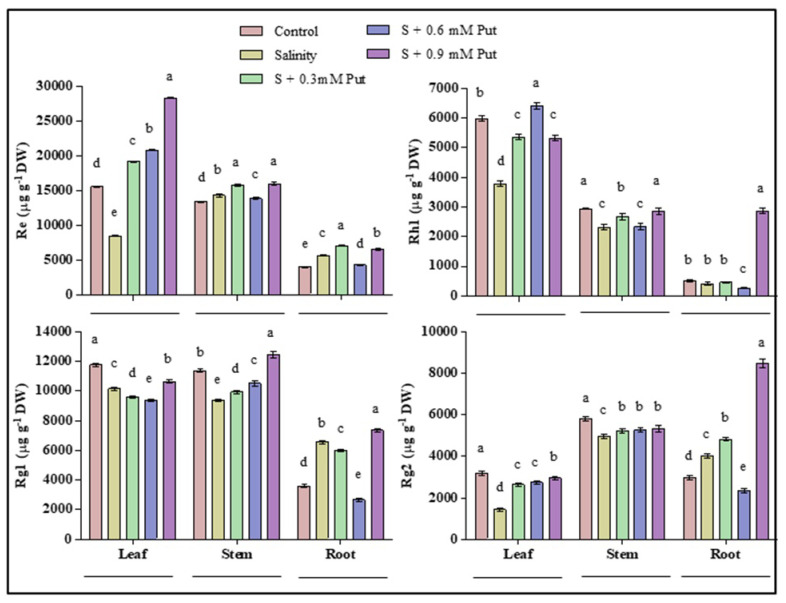
Effect of putrescine on ginsenosides (PPT type) in leaf, stem, and root of ginseng seedlings under salinity stress condition. Different letters indicate significant differences (*p* ≤ 0.05) among the treatments within each parameter. Each value represents the mean ± SD (*n* = 3).

The effects of salinity and application of Put in different concentrations protopanaxatriol (PPT) types ginsenosides in leaves, stems, and roots were shown in Figure 6. Rh1 was recorded as the main ginsenoside in both leaves and stems, where it reduced under salinity stress condition. Re, Rg1, and Rg2 were recorded as the main ginsenosides in all parts—i.e., leaves, stems, and roots. Salinity lowered the accumulation of Rg1 and Rg2 in both leaves and stems, where Re decreased only in leaves. On the other hand, Salinity stress increased these three ginsenosides in the roots of the plants. Besides, exogenous Put application improved the accumulation of Re in leaves, stems, and roots; Rh1 in leaves and stems; Rg1 in stems; and Rg2 in leaves and roots of salinity treated plants.

As with other medicinal plants, the production of saponin-containing plants, especially their secondary metabolites, significantly depends on the geographical zone, cultivation site, and different biotic and abiotic factors in the habitat, both qualitatively and quantitatively [18]. Ginsenosides production is influenced by different abiotic stresses like temperature [18], light, [104], drought [105,106], and salinity [107]. The production of secondary metabolites may also be altered by environmental stresses [108]. In the present study, salinity stress increased the ginsenosides Rb1, Rc, Rd, and Rg3 accumulation and decreased others. Plants accumulate secondary metabolites as a response to environmental stress [109]. Ginsenosides have an adverse allelopathic effect on plants, pathogens, and pests. It also has involvement in biological tolerance mechanisms to environmental stress [110]. These results were indicating a direct influence of salinity on ginsenosides accumulation in plants. In a previous study, the accumulation of Rb1 was significantly increased in roots under chilling stress and further decreased by treating methyl jasmonate (MJ) whereas Rc and Rd did not show any response under chilling but increased in MJ treated plants. In case of PPT type, Re and Rg2 enhanced, and Rg1 decreased under chilling, whereas vice versa results were observed under MJ treatment. According to their suggestions, PPT type ginsenosides induced under abiotic stress, and PPD type ginsenosides enhanced in the plants treated by MJ [16]. In our findings, except L F2, L Rb3, and S Rb3, all other ginsenosides were increased under salinity stress. Exogeneous Put enhanced all PPD type ginsenosides in most cases except L Rg3 and S Rg3. Although salinity stress enhanced all PPT types ginsenosides in roots, but opposite results were observed in both leaves and stems. Interestingly, exogeneous Put significantly enhanced PPT types ginsenosides in most cases. Therefore, present findings suggesting that, PPD types ginsenosides enhanced by both salinity stress and exogeneous Put treatment. On the other hand, PPT types ginsenosides enhanced in roots and, reduced in leaves and stems under salinity stress condition whereas they enhanced by exogeneous Put application in all parts of the plants. However, in spite of having a little information about physiological role of ginsenosides, its defensive role is preconceived [15,111].

### 2.7. Hierarchical Clustering and PCA Analysis

**Figure 7:**
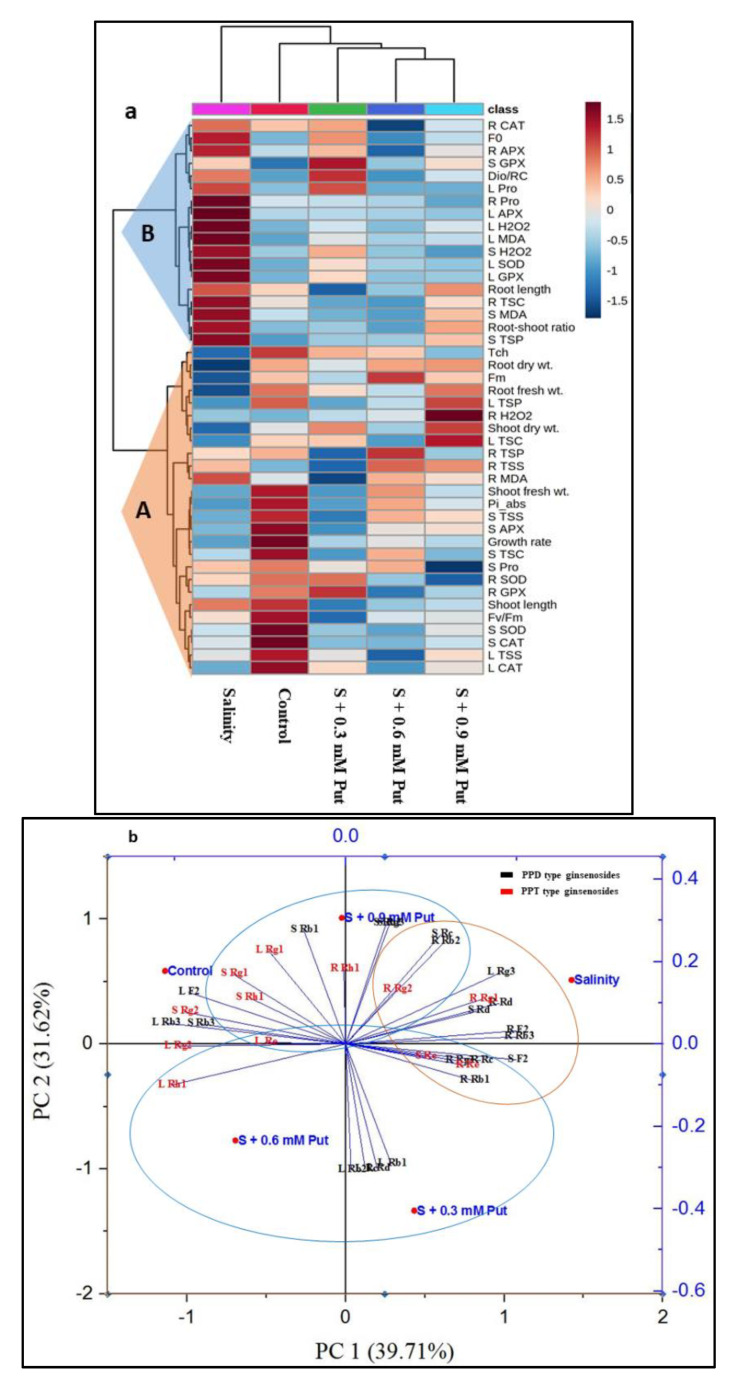
Hierarchical clustering and heatmap analysis (**a**), and principal component analysis (PCA) (**b**) to elucidate the variable treatment relationships under five treatments for 5 days. In the heatmap, the mean values of the various parameters obtained in this study were normalized and clustered. At the variable level, four separate clusters were recognized for each treatment. The color scale displays the intensity of normalized mean values of different parameters. In PCA, the lines starting from the central point of the biplots display negative or positive associations of different variables, and their proximity specifies the degree of correlation with specific treatment.

The morpho-physiological and biochemical data from ginseng seedlings under all treatments were employed to construct a heatmap, hierarchical clustering, and PCA. Hierarchical clustering grouped all the variables into two clusters (cluster-A, and cluster-B) (Figure 7a). All the variables (except R MDA) of cluster A were significantly reduced under salinity stress compared to control. Compared to salinity, Tch, R SOD, L CAT, and R GPX showed significantly higher value by applying 0.3 mM concentration of Put, whereas the variables like plant growth rate, shoot fresh weight, Fm, Fv/Fm, Pi_abs, S Pro, S TSC, S TSS, R TSP, and R TSS by 0.6 mM concentration and root fresh weight, root dry weight, shoot dry weight, L TSC, L TSS, L TSP, R H_2_O_2_, and S SOD by 0.9 mM Put concentration. In cluster B, salinity showed a higher value of the variables compared to control. On the other hand, exogenous Put at 0.3 mM concentration showed the maximum value of S GPX, Dio/RC, and L Pro. 

The PCA analysis was carried out to uncover the connection of different ginsenosides with all treatment groups (Figure 7b). The elements PC1 and PC2 together describes 71.33% of data variability. The treatment control and salinity manifested an opposite relationship where both PPD and PPT type ginsenosides from leaves, stems, and roots were segregated with respect to five treatments. In case of PPT type ginsenosides, Roots have an intimate relationship with salinity stress, where leaves and stems manifested close relationship with control and exogenous Put treatments. In case of PPD type ginsenosides, the variables L Rg3, S F2, S Rb2, S Rc, R F2, R Rb2, R Rb3, R Rc, and R Rd interlinked with salinity stress where the rest of the variables are intimately associated with control and exogenous Put treatments.

**Figure 8:**
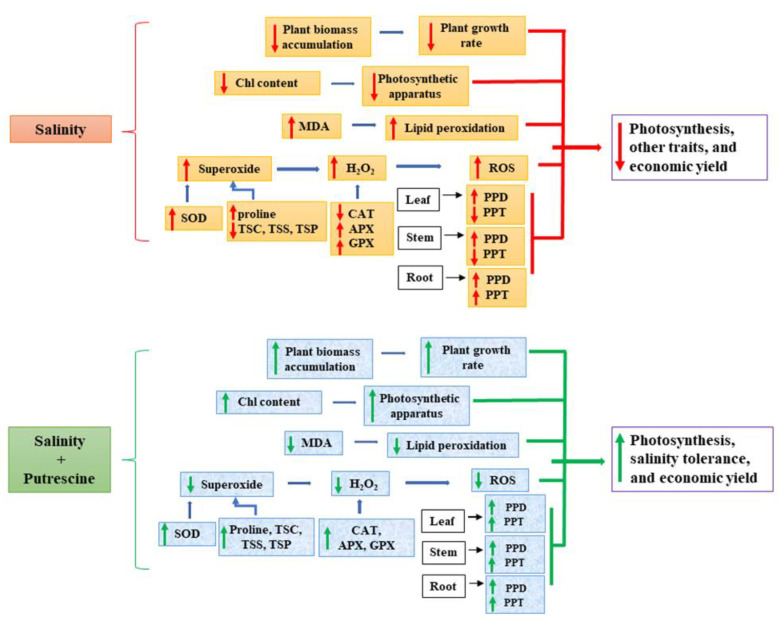
Schematic representation of salinity-induced growth inhibition and improvement of growth by exogenously Put treatment. Chl, chlorophyll; H_2_O_2_, hydrogen peroxide; ROS, reactive oxygen species; Pro, proline; TSC, total soluble carbohydrate; TSS, total soluble sugar; TSP, total soluble protein; SOD, superoxide dismutase; CAT, catalase; APX, ascorbate peroxidase; GPX, guaiacol peroxidase; MDA, malondialdehyde.

The schematic diagram (Figure 8) represents that salinity exerts its harmful effects on plants by reducing the chlorophyll content resulting in decreased other photosynthetic traits. Salinity increases superoxide, which in turn increases H_2_O_2_ content, contributing to excessive production of MDA. Salinity induces lower levels of TSC, TSS, and TSP with reduced activity of CAT enzyme. Activities of SOD, APX, and GPX are also affected by salinity to some extent, resulting in an increase in H_2_O_2_. PPD types ginsenosides increased under salinity in all parts of the plants. PPT types decreased in leaves and stems while increased in roots under salinity. In contrast, exogenous Put treatment can restore the growth of salinity-stressed plants and reduce oxidative damage. Put can maintain Chl contents, help to maintain photosynthetic traits. Exogenous Put induced increase in osmolytes like Pro, TSC, TSS, and TSP along with antioxidant enzymes like SOD, CAT, APX, and GPX maintain the optimum level of ROS. Put reduces the cell membrane damage by lowering lipid peroxidation, which was confirmed through reduced MDA. Exogenous Put increased both PPD and PPT types ginsenosides in leaves, stems, and roots. 

## 3. Materials and Methods

### 3.1. Plant Materials and Salt Treatment

The roots of ginseng (*Panax ginseng* Meyer) were placed in the germination tray (50 × 35 × 10 cm) containing a formulated substrate (commercial horticultural soil: organic manure = 3:1). The pots were then placed in a greenhouse and irrigated daily using tap water. After 23 days, uniformly grown seedlings were transferred to hydroponic containers with nutrient formulated water (Table 2). All seedlings grew under control conditions for seven days then transferred to separate hydroponic containers for control (nutrients solution only) and salinity stress condition (nutrients with 150 mM NaCl). After that, the seedlings under salinity stress were treated with Put at 0.3, 0.6, and 0.9 mM concentration levels, where control plants were treated with water only on 30 days old seedlings. Put, and water was applied to both sides of the leaves of seedlings for one time with 1% Tween-20 (*v*/*v*). After five days, the samples (youngest completely formed leaves, shoots, and roots) were collected for further analysis.

### 3.2. Determination of Plant Growth Parameters

Six plants were selected at random for data collection. The plant growth rate was calculated based on height differences between first and fifth days of treatments application. On the fifth day from the onset of treatments, seedlings were placed in an oven at 60 °C for 48 h, to determine the dry weight.

### 3.3. Determination of Chlorophyll Content and Fluorescence Parameters

Total chlorophyll of freeze-dried leaf sample was determined according to the method described by Lichtenthaler [112]. The photosynthetic fluorescence parameters were measured using a Fluor Pen FP 100 (Photon system Instruments, Czech Republic) by measuring OJIP transient under dark-adapted condition for 20 min.

### 3.4. Estimation of Osmotic Adjustment Molecules

Approximately 25 mg of freeze-dried plant material was used to estimate proline concentration. The concentration of proline was determined by following the method described by Bates [113]. The absorbance was taken at 520 nm with a UV–vis spectrophotometer (UV-1800 240 V, Shimadzu Corporation, Kyoto, Japan), and calculations were done using an appropriate proline standard curve.

To analyze TSC and TSS, a 25 mg freeze-dried sample was homogenized in 5 mL of ethanol (95%). After that, the homogenized sample was centrifuged at 5000 rpm for 10 min, and the supernatant was collected. The whole process was repeated with 70% ethanol, and the supernatant was kept in a refrigerator (4 °C) for the analysis. TSC and TSS content was determined according to the methods described by khoyerdi et al. [74] and van Handel [114], respectively.

### 3.5. Determination of Malondialdehyde and H_2_O_2_ Content

The fresh plant samples (200 mg) were macerated in 0.1% trichloroacetic acid (5 mL). The homogenate was centrifuged at 12,000× *g* for 10 min at 4 °C, and the supernatant was stored at 4 °C for analysis. Lipid peroxidation was determined by estimating the malondialdehyde (MDA) content in the leaves, stems and roots of the ginseng seedlings. The procedure for the estimation of MDA and H_2_O_2_ was described in our previously published article [8]. 

### 3.6. Determination of Antioxidant Enzyme Activities

Samples (leaves, stems, and roots) from all treated plants were collected and immersed immediately in liquid nitrogen and stored at −80 °C until use. A 200 mg sample was homogenized in 5 mL of 50 mM sodium phosphate buffer solution (pH 7.8) using a pre-chilled mortar and pestle, then centrifuged at 15,000× *g* for 20 min at 4 °C. After collecting the supernatant, the enzyme extract was stored at 4 °C for analysis. The activity of superoxide dismutase (SOD; EC 1.15.1.1) was estimated by the method described earlier [8]. The guaiacol peroxidase (GPX; EC 1.11.1.7) and catalase (CAT; EC 1.11.1.6) activities were determined according to Zhang [115]. The activity of ascorbate peroxidase (APX; EC 1.11.1.11) was assayed by the method developed earlier [116]. 

### 3.7. Estimation of Protein Amount

For all enzymatic activities calculation, protein content was determined spectrophotometrically at 595 nm by the method of Bradford [117].

### 3.8. Analysis of Ginsenosides by High-Performance Liquid Chromatography

The freeze-dried ginseng sample was crushed to 80 mesh, and 100 mg of leaves, stems and roots were measured, and 2 mL of 70% methanol (Mallinckrodt Baker Inc., Phillipsburg, NJ, USA) was added. Then, it was extracted by sonicator at 50 °C for 40 min. The extract was filtered through Whatman (Kent, England) no. 6 filter paper and concentrated with a rotary evaporator (Eyela, Tokyo, Japan) in a 30 ℃ water bath. The solid content was dissolved in 70% methanol to make 5000 ppm (5000 ug/mL).

The HPLC separation was carried out by the method developed by Lee et al. (2016) [118] with slight modification on a Shimadzu LC-20AT HPLC system. At first, 3 mL MeOH and 3 mL dd-H_2_O was slowly eluted with the Sep-pak Plus C18 cartridge (Waters Co., Milford, MA, USA) for conditioning. 1 mL of the extract was loaded into the cartridge and then eluted with 10 mL dd-H_2_O (Mallinckrodt Baker Inc., Phillipsberg, NJ, USA), and it was dried by flowing ultra-high purity grade nitrogen gas (Milsung Industrial Gas Co., Ltd., Hwaseong, Korea). The cartridge was treated with 2 mL MeOH to elute the ginsenoside component which was filtrated through a 0.45 μm membrane filter before HPLC analysis. Distilled water (JT baker) was used as solvent A and acetonitrile (99.8%; JT baker) as solvent B for mobile phase with a flow rate of 1.0 mL/min and the following gradient: initial conditions 72% A, 28% B; 0–10 min 72% A, 28% B; 10–45 min 60% A, 40% B; 45–47 min 100% A, 0% B; 47–68 min 100% A, 0% B; 68–70 min 72% A, 28% B; 70–75 min 72% A, 28% B. The oven temperature was maintained at 40 ℃ throughout the separation process, and injected volume was 20 µL.

### 3.9. Statistical Analysis

All the data provided in the tables and figures are mean ± standard deviation. The data presented in the table and graphs were analyzed by SAS 9.4 (SAS Institute Inc., Cary, NC, USA) program following complete randomized design (CRD), and the mean differences were compared by the least significant (LSD) test. *p*-values ≤ 0.05 were significant. The heatmap and clustering analysis were prepared from normalized mean values using the MetaboAnalyst 4.0 (www.metaboanalyst.ca, accessed on 21 June 2021). Hierarchical cluster analysis was conducted using the Euclidean distance algorithm. The principal component analysis (PCA) was carried out using the OriginLab 10.0 software (OriginLab, Northampton, MA, USA).

## 4. Conclusions

The exogenous Put significantly reduced H_2_O_2_ production and MDA content through maintaining the balance of osmolytes and antioxidant enzymes, which triggered the protection against cellular damage of ginseng sprouts. The recompensating of total chlorophyll and chlorophyll fluorescence from saline stress may be due to the catabolic activity of putrescine that protected the plants from stress-derived damage that maintained the morpho-physiological activities (Figure 8). It is plausible that the ROS-scavenging and cell-protecting properties of Put enhanced the salt tolerance in ginseng seedlings. Hence, exogenous application of Put can effectively improve the growth and adaptation of ginseng plants under saline condition. Overall, PPD types ginsenosides enhanced by both salinity stress and exogenous Put treatment. On the other hand, PPT types ginsenosides enhanced in roots and reduced in leaves and stems under salinity stress condition, whereas they enhanced by exogenous Put application in all parts of the plants, as evidenced by PCA analysis. However, further investigations at the genetic and molecular levels are crucial to discover the exact potentiality of Put in salinity-adaptive mechanisms of ginseng sprouts and the role of PPD and PPT ginsenosides in stress tolerance.

## Figures and Tables

**Table 1:** Effect of foliar application of putrescine (Put) on the plant growth rate (GR), shoot length (SL), root length (RL), root-shoot ratio (RSR), shoot fresh weight (SFW), shoot dry weight (SDW), root fresh weight (RFW), and root dry weight (RDW) of ginseng sprouts grown under salinity stress.

Treatment	Growth Rate (cm/day)	Shoot Length (cm)	Root Length (cm)	Root-Shoot Ratio	Shoot Fresh Weight (gm)	Shoot Dry Weight (gm)	Root Fresh Weight (gm)	Root Dry Weight (gm)
Control	0.321 ± 0.11 a	11.72 ± 1.17 a	11.72 ± 1.48	1.00 ± 0.12	0.502 ± 0.10 a	0.082 ± 0.01	0.967 ± 0.14 a	0.129 ± 0.02 a
Salinity	0.054 ± 0.06 c	10.52 ± 1.43 ab	11.73 ± 2.75	1.15 ± 0.38	0.326 ± 0.02 c	0.074 ± 0.02	0.730 ± 0.08 b	0.087 ± 0.01 b
S + 0.3 mM Put	0.112 ± 0.10 bc	9.97 ± 1.65 b	10.77 ± 2.05	1.10 ± 0.24	0.370 ± 0.05 bc	0.087 ± 0.01	0.987 ± 0.16 a	0.127 ± 0.02 a
S + 0.6 mM Put	0.162 ± 0.04 b	11.30 ± 1.17 ab	12.45 ± 2.76	1.12 ± 0.29	0.517 ± 0.08 a	0.086 ± 0.01	1.012 ± 0.20 a	0.146 ± 0.02 a
S + 0.9 mM Put	0.116 ± 0.07 bc	10.73 ± 0.59 ab	12.98 ± 2.45	1.21 ± 0.22	0.413 ± 0.05 b	0.086 ± 0.01	1.029 ± 0.19 a	0.136 ± 0.03 a
LSD_0.05_	0.092	1.49	NS	NS	0.078	NS	0.193	0.027

Different letters indicate significant differences (*p* ≤ 0.05) among the genotypes within each parameter. Values are expressed as mean ± SD (*n* = 6).

**Table 2:** Nutrient solution.

Chemical Name	A Tank (50 L) *	B Tank (50 L)
Ca(NO_3_)	1.5 kg	
KNO_3_	3.79 kg	3.79 kg
(NH_4_)_2_HPO_4_		1.6 kg
MgSO_4_		4.3 kg
K_2_SO_4_		
Fe-EDTA	460 g	
MnSO_4_		30.8g
H_3_BO_3_		57.2 g
ZnSO_4_		3.6 g
CuSO_4_		1.3 g
(NH_4_)_6_Mo_7_O_24_.4H_2_O		0.4 g

* Solution of Tank A and Tank B were subjected to mixed to maintain a E.C. 0.4 dSm^−2^, pH. 6.0.

## Data Availability

Not applicable.
